# Early alkaline phosphatase dynamics as biomarker of survival in metastatic castration-resistant prostate cancer patients treated with radium-223

**DOI:** 10.1007/s00259-021-05283-6

**Published:** 2021-03-08

**Authors:** Maarten J. van der Doelen, Agnes Stockhaus, Yuanjun Ma, Niven Mehra, Jeffrey Yachnin, Winald R. Gerritsen, Sten Nilsson, Inge M. van Oort, Anders Ullén

**Affiliations:** 1 Department of Urology, Radboud University Medical Center, Geert Grooteplein Zuid 10, 6525 GA Nijmegen, The Netherlands; 2 Department of Medical Oncology, Radboud University Medical Center, Nijmegen, The Netherlands; 3 Department of Oncology-Pathology, Mälarsjukhuset, Eskilstuna, Sweden; 4 Department of Oncology-Pathology, Karolinska Institutet, Stockholm, Sweden; 5 Department of Pelvic Cancer, Genitourinary Oncology and Urology Unit, Karolinska University Hospital, Stockholm, Sweden

**Keywords:** Alkaline phosphatase, Biomarker, Bone metastases, Castration-resistant prostate cancer, Radium-223, Prognostic variables

## Abstract

**Purpose:**

Radium-223 is a life-prolonging therapy for castration-resistant prostate cancer (CRPC) patients with symptomatic bone metastases. However, validated biomarkers for response monitoring are lacking. The study aim was to investigate whether early alkaline phosphatase (ALP) dynamics after the first radium-223 injection can act as surrogate marker for overall survival (OS).

**Methods:**

This retrospective multicenter study included consecutive CRPC patients treated with radium-223. Patients were divided into four subgroups based on baseline ALP level (normal/elevated) and early ALP response, defined as ≥10% ALP decrease after the first radium-223 injection. Primary endpoint was OS among the subgroups. Secondary endpoints included time to first skeletal-related event, time to ALP progression, and treatment completion rate.

**Results:**

A total of 180 patients were included for analysis. Median OS was 13.5 months (95% confidence interval 11.5–15.5). Patients with elevated baseline ALP without ALP response after the first injection had significantly worse OS when compared to all other patients (median OS 7.9 months versus 15.7 months, hazard ratio 2.56, 95% confidence interval 1.73–3.80, *P* < 0.001). Multivariate analysis demonstrated that elevated baseline ALP without ALP response after the first injection, the number of prior systemic therapies, baseline LDH level, and baseline ECOG performance status were prognostic factors of OS. Patients with elevated baseline ALP without ALP response after the first injection had significantly shorter times to ALP progression and first skeletal-related event, and more frequently discontinued radium-223 therapy when compared to other patients.

**Conclusion:**

Early treatment–induced changes in ALP after one radium-223 injection were associated with OS in metastatic CRPC patients.

## Introduction

Radium-223 is an alpha emitter that selectively binds to areas of increased bone turnover in bone metastases and emits high-energy alpha particles of short range, causing double-strand DNA breaks [1]. Based on significant survival benefit in the pivotal phase 3 ALSYMPCA trial, radium-223 is a registered treatment option for castration-resistant prostate cancer (CRPC) patients with symptomatic bone metastases and without any known visceral metastases [2].

Approximately 90% of CRPC patients eventually develop bone metastases [3, 4]. In these patients, serum total alkaline phosphatase (ALP) levels are often elevated and, in the absence of extensive liver disease, these levels reflect osteoblastic activity and the extent of disease [5]. This is of clinical importance, since elevated ALP levels are associated with the occurrence of skeletal-related events (SREs), independent of therapy [6, 7]. In addition, several retrospective series have reported that elevated baseline ALP levels were associated with inferior overall survival (OS) in patients treated with radium-223 [8–11].

In treatment responders, ALP has previously been shown to decrease already 4 weeks after initiation of radium-233 and may continue to decrease until the end of treatment [9]. Although the implication of baseline ALP levels on outcome has been studied, studies on ALP dynamics during and after radium-223 treatment are limited, and the utility of changes in ALP as a biomarker has to be explored [5]. In the ALSYMPCA trial, 47% of the patients experienced ≥30% ALP reduction during therapy, and real-world studies have described ≥25% ALP decrease in 50–62% of patients after initiation of radium-223 treatment [10, 12–14]. Post hoc analyses from the ALSYMPCA trial suggested that an ALP response of >30% at week 12 of therapy is associated with prolonged survival and longer time to first symptomatic skeletal event [9, 15]. Similar findings have been described in two small retrospective series of patients treated with radium-223, where OS was found to be significantly longer for patients with ≥30% ALP reduction during therapy when compared to ALP non-responders [10, 14]. However, there are currently no early biomarkers to predict overall survival during treatment with radium-223 in daily practice. The aim of this study was to investigate the prognostic value of early ALP dynamics during radium-223 therapy in patients with metastatic CRPC.

## Methods

### Study design, setting, and participants

This was a multicenter retrospective cohort study including consecutive patients treated with standard dosing of radium-223 at Karolinska University Hospital between October 2012 and December 2016, and at Radboud University Medical Center between September 2013 and January 2018. All patients were treated with radium-223 according to the original label, prior to the EMA label change of July 2018. The study was approved by the local medical ethics committees and both hospital review boards. The regulations of the Helsinki declaration were followed. Inclusion criteria were histological confirmed prostate cancer, CRPC as defined by the European Association of Urology guidelines, radiological evidence of bone metastases on bone scintigraphy, and at least one administered injection of radium-223 [16]. Patients who were concomitantly treated with abiraterone or enzalutamide were excluded.

### Data collection and follow-up

The medical records of patients were reviewed to collect demographics and clinical characteristics, including Gleason score and tumor staging, treatments and SREs prior to radium-223 therapy, use of analgesics at baseline, baseline Eastern Cooperative Oncology Group (ECOG) performance status, the use of bone health agents (denosumab or bisphosphonates) during treatment with radium-223, the number of radium-223 injections, biochemical parameters (ALP, lactate dehydrogenase (LDH), and prostate-specific antigen (PSA)), hematological parameters (hemoglobin and platelet count), date of first SRE after radium-223 initiation, and date of death. All patients were followed until death or November 1, 2020.

### Subgroup categorization

Elevated ALP was defined as baseline ALP level above the upper limit of normal of 115 U/L (1.9 ukat/L), according to institutional criteria. ALP response from baseline was defined as ≥10% ALP decline from baseline, measured 3–4 weeks after the first radium-223 injection. Non-response was classified as an ALP change of <10% or no decrease from baseline after the first radium-223 injection. The 10% ALP response cut-off was chosen because a less pronounced ALP response at an earlier time point was expected when compared to the ≥30% ALP reduction outcome measure at week 12 of therapy that was applied in the ALSYMPCA trial [2]. Moreover, the 10% ALP cut-off was used previously in a retrospective cohort study by Dizdarevic et al. [10]. Based on baseline ALP levels and ALP response from baseline, the cohort was divided in four pre-specified subgroups. Subgroups 1 and 2 consisted of patients who had normal baseline ALP levels, with and without ALP response, respectively. Similarly, subgroups 3 and 4 consisted of patients with elevated baseline ALP levels, with and without subsequent ≥10% ALP decline after the first radium-223 injection, respectively.

### Study endpoints

The primary study endpoint was OS. OS was defined as time from first radium-223 injection until death from any cause or last recorded date of follow-up. We analyzed OS in the pre-defined subgroups and evaluated prognostic factors associated with OS. Secondary endpoints included biomarker dynamics before, during, and after radium-223 therapy, the time to ALP progression, the time to first SRE, and the number of administered radium-223 injections.

ALP levels were captured 12, 9, 6, and 3 months prior to radium-223 treatment, every 4 weeks during radium-223 treatment and 3, 6, 9, and 12 months after the last radium-223 injection. Likewise, PSA levels were recorded during and after radium-223. In addition to ALP response of 10% after the first radium-223 injection, we also evaluated changes in PSA and ALP, calculated as maximal decline from baseline during radium-223 therapy, with 30% cut-off, according to the ALSYMPCA study definition [2]. Normalization of ALP was defined as an ALP level below 115 U/L in patients with elevated ALP levels at baseline. ALP progression was defined as an increase of ≥25% from baseline in patients with no decrease from baseline, or as an increase of ≥25% above the nadir, according to the ALSYMPCA study criteria [2]. In case a next systemic therapy was started prior to ≥25% increase of ALP above the nadir, no ALP progression was documented and the patient was censored. SREs were defined as surgery or radiotherapy to the bone, spinal cord compression, and pathological fractures, according to Prostate Cancer Working Group 3 criteria [17].

### Statistical analysis

Descriptive statistical methods were used to characterize the cohort. To compare the subgroups, the chi-square and Mann-Whitney *U* tests were used for categorial variables and continuous variables, respectively. Kaplan-Meier statistics were used to calculate time-to-event data. Univariate and multivariate Cox proportional hazard regression models were used to compare time-to-event distributions between the pre-specified subgroups and to assess the prognostic significance of baseline variables, presented as hazard ratios (HRs) with 95% confidence intervals (CIs). In multivariate models, forward selection and backward elimination to add or remove covariates were used. We adjusted for the following covariates at the start of radium-223 treatment: age and Gleason score at diagnosis, time from development of CRPC to initiation of radium-223 therapy, number of therapies before radium-223 initiation, ECOG performance status, and baseline hemoglobin, LDH, and PSA levels. The covariates LDH and PSA were log transformed due to distribution skewness. All statistical tests were two-sided, with *P* values of <0.05 considered to be statistically significant.

## Results

### Patient cohort

**Fig. 1:**
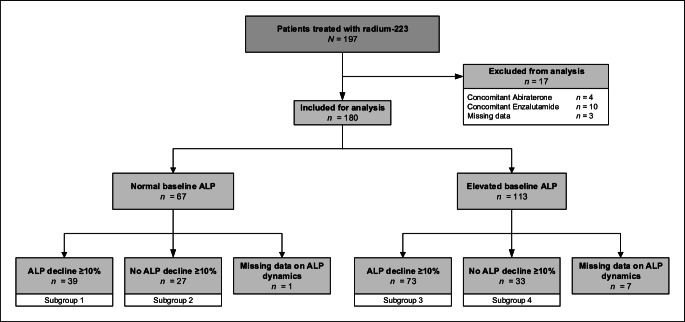
Consort diagram of the study population, including subgroup categorization based on ALP dynamics after the first injection of radium-223

In total, 197 patients with metastatic CRPC were treated at both centers during the study period. Seventeen patients were excluded from further analysis due to concomitant therapies or missing data (Fig. 1). Therefore, 180 patients were included for analysis. Baseline demographics and clinical characteristics of the total cohort and patient subgroups are shown in Table 1. Patients received a median of two prior systemic therapies, in addition to androgen deprivation therapy, for CRPC. One hundred twenty-eight (71%) patients were previously treated with abiraterone or enzalutamide and 113 (63%) patients underwent prior taxane-based chemotherapy, either upfront in hormone-sensitive (4%) or in castration-resistant (96%) state. Ninety (50%) patients had a prior SRE and bone health agents were given prior to or during radium-223 therapy in half of the patient cohort. Although bone health agents were more frequently used in patients with normal baseline ALP levels (56%) when compared to patients with high baseline ALP levels (46%), there was no statistically significant difference between the subgroups (*P =* 0.193).

**Table 1:** Baseline patient demographics and clinical characteristics

	Complete cohort (*N* = 180)			Subgroups 1–3 (*N* = 139)			Subgroup 4 (*N* = 33)			*P* value
	*n*	Median [IQR] or *n* (%)		*n*	Median [IQR] or *n* (%)		*n*	Median [IQR] or *n* (%)		
Age at start of radium-223 [y]	180	71	[66–76]	139	71	[67–76]	33	69	[62–73]	**0.030**
Initial tumor staging										
Localized prostate cancer	180	90	(50.0)	139	70	(50.4)	33	13	(39.4)	0.257
Metastatic prostate cancer	180	90	(50.0)	139	69	(49.6)	33	20	(60.6)	
ISUP 1 (Gleason score ≤ 6)	148	10	(6.8)	113	9	(8.0)	30	1	(3.3)	0.567
ISUP 2–3 (Gleason score 7)	148	40	(27.0)	113	30	(26.5)	30	10	(33.3)	
ISUP 4–5 (Gleason score ≥ 8)	148	98	(66.2)	113	74	(65.5)	30	19	(63.3)	
Time CRPC to radium-223 [mo]	180	23.2	[12.0–37.9]	139	25.3	[12.0–38.0]	33	21.0	[10.7–35.0]	0.323
Prior registered therapies for CRPC										
Median number of therapies	180	2	[1-3]	139	2	[1,2]	33	2	[1-3]	**0.031**
None	180	35	(19.4)	139	29	(20.9)	33	4	(12.1)	0.329
Docetaxel^1^	180	109	(60.6)	139	82	(59.0)	33	24	(72.7)	0.145
Cabazitaxel^2^	180	46	(25.6)	139	30	(21.6)	33	12	(36.4)	0.076
Abiraterone	180	95	(52.8)	139	75	(54.0)	33	16	(48.5)	0.571
Enzalutamide	180	52	(28.9)	139	31	(22.3)	33	18	(54.5)	**<0.001**
Opioid use	180	79	(43.9)	139	52	(37.4)	33	21	(63.6)	**0.006**
Prior SRE	180	90	(50.0)	139	72	(51.8)	33	14	(42.4)	0.333
Bone health agent use										
None	177	89	(50.3)	136	68	(50.0)	33	17	(51.5)	0.859
Denosumab	177	48	(27.1)	136	39	(28.7)	33	8	(24.2)	
Bisphosphonates	177	40	(22.6)	136	29	(21.3)	33	8	(24.2)	
ECOG performance status										
ECOG 0	172	91	(52.9)	134	74	(55.2)	31	15	(48.4)	0.784
ECOG 1	172	64	(37.2)	134	48	(35.8)	31	13	(41.9)	
ECOG 2–3	172	17	(9.9)	134	12	(9.0)	31	3	(9.7)	
Hemoglobin (g/dL)	180	12.3	[11.4–13.2]	139	12.6	[11.6–13.4]	33	11.8	[10.5–12.6]	**0.001**
Hb ≥ 10	180	168	(93.3)	139	133	(95.7)	33	28	(84.8)	**0.022**
Hb < 10	180	12	(6.7)	139	6	(4.3)	33	5	(15.2)	
Platelet count (× 10^9^/L)	180	244	[206–289]	139	249	[208–289]	33	241	[198–295]	0.616
PSA level (μg/L)	179	166	[59–417]	139	130	[54–400]	32	249	[82–499]	0.125
ALP level (U/L)	180	156	[95–264]	139	125	[85–228]	33	216	[145–348]	**<0.001**
ALP < 115	180	67	(37.2)	139	66	(47.5)	33	0	(0.0)	**<0.001**
ALP ≥ 115	180	113	(62.8)	139	73	(52.5)	33	33	(100.0)	
LD level (U/L)	166	233	[198–302]	129	219	[198–282]	29	274	[210–392]	**0.013**
LD < 250	166	96	(57.8)	129	82	(63.6)	29	12	(41.1)	**0.028**
LD ≥ 250	166	70	(42.2)	129	47	(36.4)	29	17	(58.6)	

^1^Docetaxel was administered as upfront chemotherapy in hormone-sensitive prostate cancer in four patients (2.2%)

^2^Four patients (2.2%) received cabazitaxel chemotherapy without prior docetaxel chemotherapy

*ALP*, alkaline phosphatase; *CRPC*, castration-resistant prostate cancer; *ECOG*, Eastern Cooperative Oncology Group; *IQR*, interquartile range; *ISUP*, International Society of Urological Pathology; *LD*, lactate dehydrogenase; *mo*, months; *PSA*, prostate-specific antigen; *SRE*, skeletal related event; *y*, years

Bold = *p* value < 0.05

The median ALP level at baseline was 156 U/L (interquartile range 95–264). Overall, 67 (37%) patients had normal ALP levels prior to treatment and 113 (63%) patients had elevated baseline ALP levels. Data on ALP dynamics after the first injection were missing in eight (4%) patients. Hence, data on ALP dynamics were available for analysis in 66 patients with normal baseline ALP levels and 106 patients with elevated baseline ALP levels. One hundred twelve (62%) patients experienced ≥10% ALP decrease after the first radium-223 injection. Subgroups 1, 2, 3, and 4 included 39, 27, 73, and 33 patients, respectively (Fig. 1). The patients in subgroup 4 were significantly younger at time of radium-223 initiation, used opioids more frequently, were more heavily pretreated, and had significantly lower baseline hemoglobin levels and higher LDH levels (Table 1).

### ALP dynamics and overall survival

**Fig. 2:**
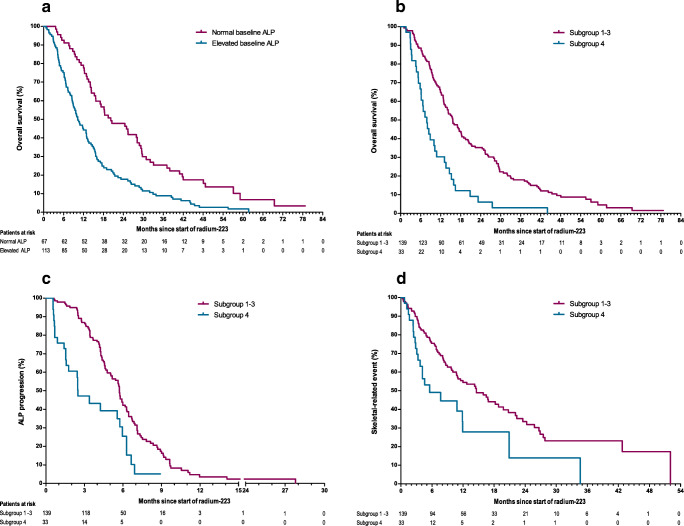
Kaplan-Meier curves. **a** Overall survival, comparing the patients with normal baseline ALP levels (subgroups 1–2) to patients with elevated baseline ALP levels (subgroups 3–4). **b** Overall survival, comparing the patients in subgroups 1–3 to the patients with elevated baseline ALP without ≥10% ALP decline after the first injection (subgroup 4). **c** Time to ALP progression, comparing the patients in subgroup 1–3 to patients with elevated baseline ALP without ≥10% ALP decline after the first injection (subgroup 4). **d** Time to first skeletal-related event, comparing the patients in subgroup 1–3 to patients with elevated baseline ALP without ≥10% ALP decline after the first injection (subgroup 4)

At time of analysis, 172 (96%) patients had deceased. The median follow-up was 13.6 (range 1–79) months. The median OS for the total study population was 13.5 months (95% CI 11.5–15.5). OS data for the total cohort and subgroups are shown in Table 2. Patients with normal baseline ALP levels (subgroups 1–2) had significantly longer OS when compared to patients with elevated baseline ALP levels (subgroups 3–4; median OS 19.5 months versus 10.8 months, HR 2.08, 95% CI 1.51–2.86, *P* < 0.001, Fig. 2a). Furthermore, patients with elevated baseline ALP without ≥10% ALP decline after the first injection (subgroup 4) had significantly worse OS than the patients in subgroups 1–3 (median OS 7.9 months versus 15.7 months, HR 2.56, 95% CI 1.73–3.80, *P* < 0.001, Fig. 2b). This association remained statistically significant after adjusting for baseline covariates (HR 2.15, 95% CI 1.35–3.43). The final multivariate model selected subgroup 4, baseline LDH level, baseline ECOG performance status, and the number of prior systemic therapies as prognostic factors of OS (Table 3).

**Table 2:** Overall survival data for the total cohort and the pre-specified subgroups

		*N* (%)	Median survival(months)	95% CI	*P* value
Total cohort		180 (100)	13.5	11.5–15.5	
Subgroup					**<0.001**
1	Normal baseline ALP, ≥10% decline after first injection	39 (21.7)	25.4	13.4–37.3	
2	Normal baseline ALP, <10% decline after first injection	27 (15.0)	18.2	11.4–24.9	
3	Elevated baseline ALP, ≥10% decline after first injection	73 (40.6)	12.9	10.5–15.3	
4	Elevated baseline ALP, <10% decline after first injection	33 (18.3)	7.9	5.9–9.9	
	Missing ALP dynamics	8 (4.4)	4.2	2.2–6.2	
Subgroups 1–2		66 (36.7)	19.5	11.8–27.1	**<0.001**
Subgroups 3–4		106 (58.9)	10.8	8.2–13.4	
Subgroups 1–3		139 (77.2)	15.7	13.0–18.4	**<0.001**
Subgroup 4		33 (18.3)	7.9	5.9–9.9	

*ALP*, alkaline phosphatase; *CI*, confidence interval

Bold = *p* value < 0.05

**Table 3:** Multivariable Cox proportional hazard analysis of overall survival

	HR	95% CI	*P* value
Age	0.99	0.97–1.02	0.65
Time CRPC to start radium-223	0.99	0.98–1.00	0.08
Initial tumor Gleason score			
Gleason score ≤ 7	Ref		
Gleason score 8–10	0.71	0.47–1.07	0.10
Baseline hemoglobin	0.89	0.76–1.04	0.15
Baseline PSA (log transformed)	1.08	0.97–1.21	0.17
Baseline LDH (log transformed)	1.80	1.17–2.77	**<0.01**
ALP dynamics during radium-223			
Subgroups 1–3	Ref		
Subgroup 4	2.15	1.35–3.43	**<0.01**
Baseline ECOG performance status			
0–1	Ref		
≥2	3.85	1.94–7.64	**<0.01**
Number of prior CRPC therapies			
0	Ref		
≥1	2.46	1.45–4.17	**<0.01**

*ALP*, alkaline phosphastase; *CI*, confidence interval; *CRPC*, castration-resistant prostate cancer; *ECOG*, Eastern Cooperative Oncology Group; *HR*, hazard ratio; *LDH*, lactate dehydrogenase; *PSA*, prostate specific antigen; *Ref*, reference

Bold = *p* value < 0.05

### Biomarker dynamics

**Fig. 3:**
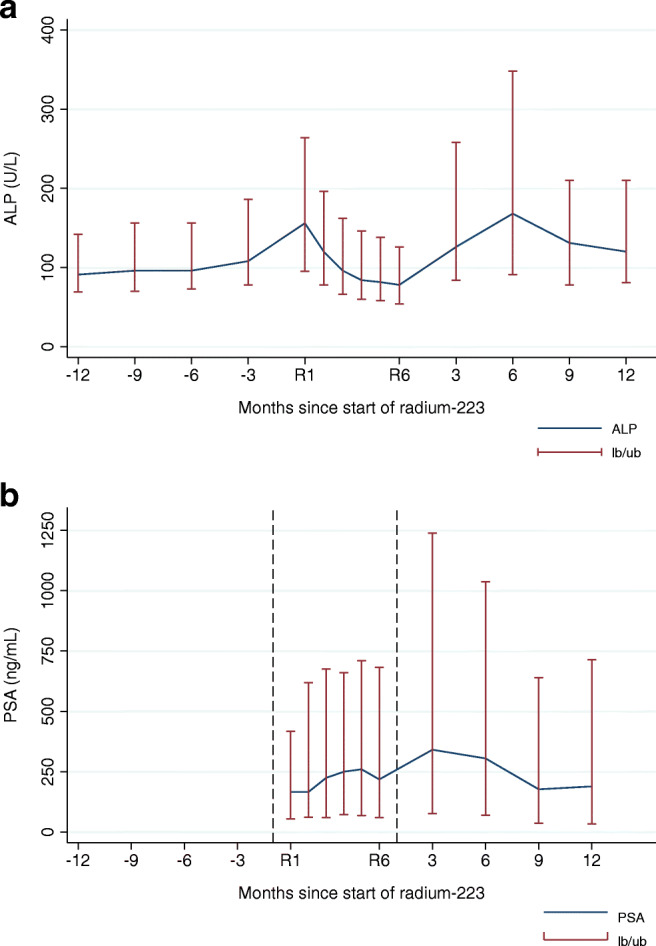
Biomarker dynamics. **a** ALP dynamics before, during, and after treatment with radium-223 (median, interquartile range). **b** PSA dynamics during and after treatment with radium-223 (median, interquartile range). R1; radium-223 injection 1; R6 radium-223 injection 6

Of the 174 patients with available data on ALP response during therapy, 108 (62%) patients experienced ≥30% ALP decrease during radium-223 therapy. Forty-six (27%) patients already showed ≥30% ALP decrease after the first radium-223 injection. The response definition of ≥10% ALP decrease after the first injection correlated with the observed ≥30% ALP decrease throughout therapy in 80% of the patients. Out of 107 patients with elevated baseline ALP levels and available data on ALP dynamics, normalization of ALP during therapy occurred in 51 (48%) patients. Figure 3a shows the change in ALP level at 12, 9, 6, and 3 months before radium-223, at each injection of radium-223 and 3, 6, 9, and 12 months after radium-223.

One hundred forty-four (80%) patients developed ALP progression before initiation of a subsequent systemic therapy. The median time to ALP progression was 5.7 months (95% CI 5.0–6.3). Patients in subgroup 4 had significantly shorter time to ALP progression when compared to all other patients (2.5 months versus 5.7 months, HR 2.30, 95% CI 1.49–3.56, *P* < 0.001, Fig. 2c).

In the cohort, the median PSA level at baseline was 166 μg/L (interquartile range 59–417). A 30% or greater reduction in PSA level during therapy was achieved in 29 (17%) patients. Of these PSA responding patients, 21 (72%) patients had ≥10% ALP decrease after the first radium-223 injection and 25 (86%) patients achieved ≥30% ALP decrease during radium-223 therapy. Figure 3b shows the change in PSA level at each radium-223 injection and 3, 6, 9, and 12 months after the last radium-223 injection.

### Treatment completion

Overall, the median number of administered injections was six and 110 (61%) patients completed radium-223 therapy. Only 39% of the patients in subgroup 4 had completed radium-223 therapy, compared to 68% of the patients in the other subgroups. The median number of administered injections was six in subgroups 1–3 and four in subgroup 4 (*P* < 0.001). Of the eight patients with missing data on ALP dynamics, four (50%) patients received just one radium-223 injection.

### Skeletal-related events

During or after radium-223, 106 (59%) patients experienced at least one SRE. In 51 (48%) of these patients, an SRE occurred within 6 months after initiation of radium-223 therapy. The median time to the first SRE was 12.0 months (95% CI 8.3–15.7). Patients in subgroup 4 had a significantly shorter time to occurrence of the first SRE when compared to all other patients (5.6 months versus 14.6 months, HR 2.01, 95% CI 1.27–3.32, *P* = 0.003, Fig. 2d). In patients with an elevated baseline ALP, the median time to SRE was significantly longer when patients experienced ≥10% ALP decline after the first injection (subgroup 3) when compared to the patients without such ALP reduction (subgroup 4) (14.4 months versus 5.6 months, HR 1.82, 95% CI 1.07–3.09, *P* = 0.027).

## Discussion

In this retrospective study of a real-world population of CRPC patients with bone metastases, we demonstrate that patients with elevated baseline ALP without subsequent ≥10% ALP decline after the first injection had significantly worse OS when compared to patients with ALP response after the first radium-223 injection. Our results are in line with a previous smaller retrospective study that showed overall survival was significantly longer in patients with ≥10% ALP reduction during therapy when compared to ALP non-responders [10]. In the present study, 84% of the patients experienced an ALP decline after the first injection, with a mean percentage change in ALP from baseline of −11%. Sixty-two percent of the patients showed ≥10% ALP decline after the first injection in this study. A post hoc analysis of the ALSYMPCA trial demonstrated a mean percentage change in ALP of −20% from baseline after the first radium-223 injection [9]. However, this post hoc analysis did not investigate an association of ALP change after the first injection with response metrics and OS.

We found that patients with normal baseline ALP levels had significantly better OS than patients with elevated baseline ALP levels. This finding correlates with previous studies that identified baseline ALP as an important prognostic factor of OS in radium-223-treated patients as well as in CRPC patients treated with other life-prolonging agents such as abiraterone and docetaxel chemotherapy [7, 9, 11, 18, 19]. In addition, low baseline ALP levels have also been associated with more frequent completion of radium-223 therapy [13, 20, 21]. These study outcomes highlight the prognostic value of baseline ALP measurements in the setting of metastatic CRPC.

Our findings suggest that ALP can serve as an early biomarker for treatment benefit, especially in patients with elevated baseline ALP levels. Based on early ALP changes, clinicians may be able to identify patients who need more close monitoring of clinical response during radium-223 therapy, since the absence of ≥10% ALP reduction may be an early indicator of treatment resistance. A post hoc analysis on data from the ALSYMPCA trial revealed that dynamic changes in ALP and lactate dehydrogenase may be useful for monitoring during treatment with radium-223, but these markers are deemed inadequate as surrogates for survival [9]. For this reason, it is not recommended to discontinue therapy solely based on changes in ALP, and other indicators of disease progression must be involved in clinical decision-making. A combinatory set of variables might have higher prognostic value. Of interest, a recent post hoc analysis of an international early access program that included 696 patients identified three risk groups based on changes in ALP and hemoglobin after three radium-223 injections. In that study, patients with ALP increase and hemoglobin decrease had significantly shorter survival than those with ALP decrease and hemoglobin increase during therapy [21].

Beyond early ALP dynamics, our multivariate analysis identified other prognostic variables of OS, including the number of prior systemic therapies and the LDH level and ECOG performance status at baseline. The prognostic value of the number of prior lines of therapies is of importance, since the European Medicines Agency recommended in July 2018 to restrict the use of radium-223 to patients who have had at least two previous treatments for metastatic CRPC or who cannot receive other treatments. This label change has impact on the treatment arsenal of metastatic CRPC patients. Due to the development of visceral metastases, impaired performance status and reduced hematological function in later disease stages, the window of opportunity to receive radium-223 therapy might be missed [4, 22, 23].

We found a significantly shorter time to the first SRE in patients with elevated baseline ALP without subsequent ≥10% ALP decline after the first injection when compared to other subjects. Of importance, bone health agents were underutilized in the present study, with only 50% of patients receiving bisphosphonates or denosumab. However, this is comparable to the use of these agents in the prospective ALSYMPCA and REASSURE studies [2, 24]. The ALSYMPCA trial has shown that radium-223 therapy was associated with a delayed time to first symptomatic SRE when compared to placebo [15]. Since the randomized phase 3 ERA-223 trial revealed that the combination of abiraterone and prednisone plus radium-223 increased the risk of bone fractures when compared with the abiraterone and prednisone plus placebo group, the prevention of SREs in metastatic CRPC patients has gained more attention [22, 25, 26]. Our findings and the outcomes of the ERA-223 trial warrant the introduction of bone health agents to prevent skeletal morbidity in patients with metastatic CRPC prior to initiation of radium-223, especially in patients with elevated baseline ALP levels, if not already started in an earlier phase of CRPC.

Our findings and the observations in previous studies indicate that bone-related parameters are strong prognostic variables for OS in CRPC with bone metastases. To date, validated liquid blood or urinary markers for monitoring of radium-223 treatment in metastatic CRPC represent an unmet medical need. Several markers of bone metabolism, including bone-specific ALP, procollagen type 1 N-terminal propeptide, procollagen type 1 C-terminal propeptide, C-telopeptide of type 1 collagen, and N-telopeptide of type 1 collagen, have been suggested as potential surrogate markers to monitor treatment with radium-223 [27–29]. Future prospective clinical studies might incorporate markers of bone turnover when evaluating radium-223 treatment.

The current study has several limitations that should be notified. Due to the retrospective nature, the data should be interpreted with caution when making decisions regarding treatment discontinuation. Selection bias might be introduced due to the exclusion of patients without follow-up ALP levels available. The missing of these data can possibly be explained by early discontinuation of radium-223 therapy due to progressive disease. Since the patients in this study were treated in two large academic hospitals, the results may differ from outcomes of patients treated in community hospitals. In addition, the majority of patients were treated in the era prior to the introduction of upfront docetaxel or abiraterone in the setting of metastatic hormone-sensitive prostate cancer. However, the study is strengthened by the multicenter design and the relatively large cohort of patients consecutively treated with radium-223, in daily practice.

In conclusion, we showed that early ALP dynamics during radium-223 therapy hold potential to serve as a treatment-predictive biomarker for OS of metastatic CRPC patients in a real-world setting. The findings in this study would benefit from further validation in prospective clinical trials where radium-223 is used according to the current market authorizations.
